# Does Method of Placental Removal or Site of Uterine Incision Repair Alter Endometritis After Cesarean Delivery?

**DOI:** 10.1155/S106474499300016X

**Published:** 1993

**Authors:** Everett F. Magann, Mark K. Dodson, Robert L. Harris, Randall C. Floyd, James N. Martin, John C. Morrison

**Affiliations:** 1 Department of Obstetrics and Gynecology University of Mississippi Medical Center Jackson MS USA; 2 Department of Obstetrics and Gynecology University of Mississippi Medical Center 2500 North State Street Jackson MS 39216-4505 USA

## Abstract

*Objective:* his investigation was undertaken to evaluate the relationship between postcesarean endometritis and (1) method of placental removal and (2) site for uterine repair.

*Methods:* This prospective, randomized study included 120 patients who underwent primary or repeat abdominal delivery for arrest of progress in labor, fetal distress, or breech presentation. Parturients were divided into four groups: I—spontaneous placental detachment, in situ uterine repair; II—spontaneous placental detachment, exteriorized uterine repair; III—manual placental removal, in situ uterine repair; and IV—manual placental removal, exteriorized uterine repair. Prophylactic antibiotics were not used.

*Results:* Endometritis was significantly increased in the manual removal/exteriorized uterine repair group versus all the other groups including the spontaneous removal in situ (group I, *P* = 0.012), the spontaneous removal/exteriorized repair group (group II, *P* = 0.034), and the manual removal/in situ repair group (group III, *P* = 0.043). Comparison of group IV (manual removal/ exteriorized repair) with the combined groups I, II, and III (spontaneous removal/in situ repair, spontaneous removal/exteriorized repair, and manual removal/in situ repair) was significantly different (*P* = 0.005). Prior to delivery, use of an internal monitoring system, skill of the operating surgeon, and type of anesthesia were similar among groups.

*Conclusions:* The findings of this investigation suggest that; when other known causes of infectious morbidity are constant, manual placental remvol in association with exteriorization for uterine repair significantly increases postcesarean endometritis.


Infectious Diseases in Obstetrics and Gynecology 1:65–70 (1993)
© 1993 Wiley-Liss, Inc.

### Does Method of Placental Removal or Site of Uterine Incision Repair Alter Endometritis After Cesarean Delivery?

**Everett F. Magann, Mark K. Dodson, Robert L. Harris, Randall C. Floyd, James N. Martin, Jr., and John C. Morrison**

*Department of Obstetrics and Gynecology, University of Mississippi Medical Center, Jackson, MS*

ABSTRACT

***Objective:*** **his investigation was undertaken to evaluate the relationship between postcesarean endometritis and (1) method of placental removal and (2) site for uterine repair.**

***Methods:*** **This prospective, randomized study included 120 patients who underwent primary or repeat abdominal delivery for arrest of progress in labor, fetal distress, or breech presentation. Parturients were divided into four groups: I—spontaneous placental detachment, in situ uterine repair; II—spontaneous placental detachment, exteriorized uterine repair; III—manual placental removal, in situ uterine repair; and IV—manual placental removal, exteriorized uterine repair. Prophylactic antibiotics were not used.**

***Results:*** **Endometritis was significantly increased in the manual removal/exteriorized uterine repair group versus all the other groups including the spontaneous removal in situ (group I, $P = 0.012$), the spontaneous removal/exteriorized repair group (group II, $P = 0.034$), and the manual removal/in situ repair group (group III, $P = 0.043$). Comparison of group IV (manual removal/exteriorized repair) with the combined groups I, II, and III (spontaneous removal/in situ repair, spontaneous removal/exteriorized repair, and manual removal/in situ repair) was significantly different ($P = 0.005$). Prior to delivery, use of an internal monitoring system, skill of the operating surgeon, and type of anesthesia were similar among groups.**

***Conclusions:*** **The findings of this investigation suggest that, when other known causes of infectious morbidity are constant, manual placental removal in association with exteriorization for uterine repair significantly increases postcesarean endometritis.** © 1993 Wiley-Liss, Inc.

KEY WORDS

Spontaneous expulsion, manual extraction, uterine position

Postpartum endometritis, defined as a maternal temperature of > 38°C on two separate occasions 6 hours apart after the first 24 hours with uterine tenderness and/or foul-smelling lochia, is a common postoperative complication of cesarean surgery. The incidence of this complication in an indigent patient population ranges between 20% and 85%.[1] A number of operative and obstetric factors are related to the development of infection following cesarean birth. Obstetric factors that are thought to contribute to the development of postoperative endometritis include: (1) the duration of labor[2,3]; (2) rupture of the membranes and the length of time between membrane rupture and operative delivery[4,5]; (3) the number of vaginal examinations[6]; (4) the use of internal fetal scalp and uterine pressure monitoring devices[7]; and (5) indigent patients regardless of race.[8] Operatives factors that have an impact on postcesarean infectious morbidity include: (1) the skill of the operating surgeon[6]; (2)

Address correspondence/reprint requests to Dr. Everett F. Magann, Department of Obstetrics and Gynecology, University of Mississippi Medical Center, 2500 North State Street, Jackson, MS 39216-4505.

Received November 18, 1992
Accepted May 5, 1993

procedure length > 1 hour[9]; (3) type of anesthesia[10]; (4) blood loss > 800 ml[9]; and (5) maternal obesity.[11]

There is a diversity of published opinion in regard to a recommended method for removal of the placenta during cesarean surgery. Should it be expelled spontaneously[12] or manually extracted?[13] Similarly, two methods of uterine incision repair are recommended, one, in situ tissue approximation to avoid trauma to the adnexa,[14] or two, uterine exteriorization to facilitate wound closure.[15] Hershey and Quilligan[16] demonstrated no increase in the incidence of postoperative infectious morbidity following cesarean delivery with external uterine repair. However, the associated method of placental removal was not addressed.

The purpose of this investigation was to evaluate the impact of the method of placental removal (spontaneous or manual) and the site of uterine repair (in situ or exteriorized) on the incidence of infectious morbidity following cesarean birth.

### MATERIALS AND METHODS

This prospective, randomized study included 120 consecutive patients who had a cesarean delivery for obstetric reasons between October 1991 and January 1992. Exclusion factors were the presence of chorioamnionitis at the time of cesarean birth, patient refusal to participate following informed consent, and antenatal treatment with steroids or insulin. A sample size and power analysis were done prior to initiating this study. It was calculated that, to reduce the rate of endometritis from 40% to 20%, 42 women would be needed in each arm of the study. After 30 women had been evaluated in each arm, an interim analysis was performed. Because a statistically significant difference was already evident, no further patients were enrolled into this study.

All patients in this study agreed to participate and signed an informed consent form that was approved by the Committee on Human Investigation at the University of Mississippi Medical Center. The informed consent made patients aware that prophylactic antibiotics would not be used so that the unique relationship between placental management and site of uterine repair could be targeted for evaluation. Study subjects were placed into one of four groups: I—spontaneous placental detachment/in situ uterine repair; II—spontaneous placental detachment/exteriorized uterine repair; III—manual placental removal/in situ uterine repair; or IV—manual placental removal/exteriorized uterine repair. Random group assignment was ensured by card selection from sealed opaque envelopes with group appointment derived from a random number table.

The population cared for by the obstetric service at the University of Mississippi Medical Center is predominantly an indigent population (79% below the 125 percentile of poverty level). The profile of patients was not significantly different between those who underwent repeat cesarean delivery without trial of labor and those who labored prior to abdominal surgery. The incidence of postcesarean endometritis was approximately 33% in this population.

The indications for operative delivery were fetal distress, arrest of progress in labor, repeat cesarean surgery, breech presentation, and severe preeclampsia without thrombocytopenia or coagulopathy. No prophylactic antibiotics were used, none of the patients were on antibiotics for urinary tract infection or group B streptococcus therapy, and the 12 patients with chorioamnionitis on antibiotics at the time of cesarean birth were excluded. Chromic suture material was employed on the uterus, polyglycolic suture on the fascia, and skin clips on each operation procedure. After delivery of the infant and according to previously determined group assignment, the placenta was either manually removed or allowed to detach spontaneously with gentle cord traction and uterine massage, and then the uterus was left in situ or exteriorized for uterine incision closure. All patients had their uterus cleansed with a gauze sponge after the placenta had been removed. All uterine incisions were closed with a double-layer closure using chromic suture. The pelvis was irrigated with saline prior to abdominal wound closure in all patients.

The diagnosis of infectious morbidity was made by (1) an elevation of the maternal temperature of > 38°C on two separate occasions 6 hours apart the first 24 hours with (2) uterine tenderness, and/or (3) a foul-smelling lochia. Once febrile morbidity was identified, complete physical examination including a pelvic examination was performed. After urine and blood but not endometrial cultures were

**TABLE 1. Demographic characteristics**

| Group | Age | Race[a] | Nulliparas/multiparas | Type of cesarean (primary/repeat) | Length of procedure (min) |
|---|---|---|---|---|---|
| I. Spontaneous in situ | 24.6 ± 4.4 | 19/7/4 | 14/16 | 22/8 | 34.6 ± 11 |
| II. Spontaneous exteriorized | 22.6 ± 4.7 | 22/7/1 | 13/17 | 24/6 | 32.5 ± 15 |
| III. Manual in situ | 25.2 ± 6.9 | 21/6/3 | 16/14 | 23/7 | 38.2 ± 8.5 |
| IV. Manual exteriorized | 22.5 ± 8.5 | 23/4/3 | 15/15 | 22/8 | 34.8 ± 10 |
| *P* value | NS | NS | NS | NS | NS |

[a] Black/Caucasian/American Indian.
NS, not significant.

obtained, patients with suspected endometritis were treated with intravenously administered triple antibiotics consisting of ampicillin, 2 g every 6 hours; gentamicin (Garamycin, Schering Corporation, Kenilworth, NJ), 1.5 mg/kg every 8 hours; and clindamycin, 900 mg every 8 hours. Endometrial cultures were not obtained because they yielded inconclusive results associated with contaminated specimens obtained transcervically. All patients were placed on triple antibiotics, and uterine cultures did not alter antibiotic management.

Surgery was primarily performed by a second-year Ob-Gyn resident with the assistance of either a fourth-year Ob-Gyn resident or maternal-fetal medicine fellow. Labor and delivery personnel acted as the scrub and circulating nurses on all surgeries. All operative procedures were performed between 0700 and 2300 hours in order that one of the two authors (E.F.M. or M.K.D.) could be present to calculate blood loss. The duration of labor, length of rupture of the membranes prior to delivery, number of vaginal examinations, and use of an internal monitoring system were recorded for all patients. The internal monitoring system consisted of a fetal scalp electrode and an open-ended, fluid-filled catheter inserted through the cervix and attached to a strain-gauge transducer. The types of abdominal and uterine incision were recorded for each operative case. Each operative procedure was assessed for length of surgery, type of anesthesia used, and amount of blood loss. The blood loss was determined by a modification of the volumetric technique of Wangensteen,[17] by which blood in the suction apparatus prior to irrigation is measured and the laparotomy pads and sponges are weighed.

Statistical analysis was performed by corrected chi-square, Fisher's exact test, and Wilcoxon test. A $P$ value of $< 0.05$ was considered statistically significant.

### RESULTS

One hundred twenty women were enrolled in this study, with 30 women assigned to each of the four arms. Due to the findings of elevated maternal temperature, leukocytosis with a left shift, and foul-smelling amniotic fluid at the time of cesarean surgery with subsequent culture confirmation of infection done at the time of cesarean section, the investigators excluded several patients from the study. These included three women from the spontaneous removal/in situ repair group (group I), four from the spontaneous removal/exteriorized repair group (group II), two from the manual removal/in situ repair group (group III), and three from the manual removal/exteriorized repair group (group IV). The demographics of age, race, gravidity, operative time, indications for cesarean section, and number of repeat cesarean operations with concurrent bilateral tubal ligation were similar among groups (Table 1). The incidence of postcesarean endometritis was 33% (36/108) overall. Endometritis occurred in repeat cesarean deliveries without labor in 15.8% (3/19), in contrast to 40% (4/10) in repeat cesarean delivery patients who experienced labor. Other deliveries without labor included 14 women with breech presentations, 3 (21.4%) of whom developed postpartum endometritis.

Excluding women with chorioamnionitis (3, 4, 2, 3, respectively, in groups I–IV) and comparing infected and noninfected women in groups I–IV by chi-square, we calculated a statistically significant difference ($P = 0.04$) with three degrees of freedom. Comparison of group IV (15 infected, 12 not

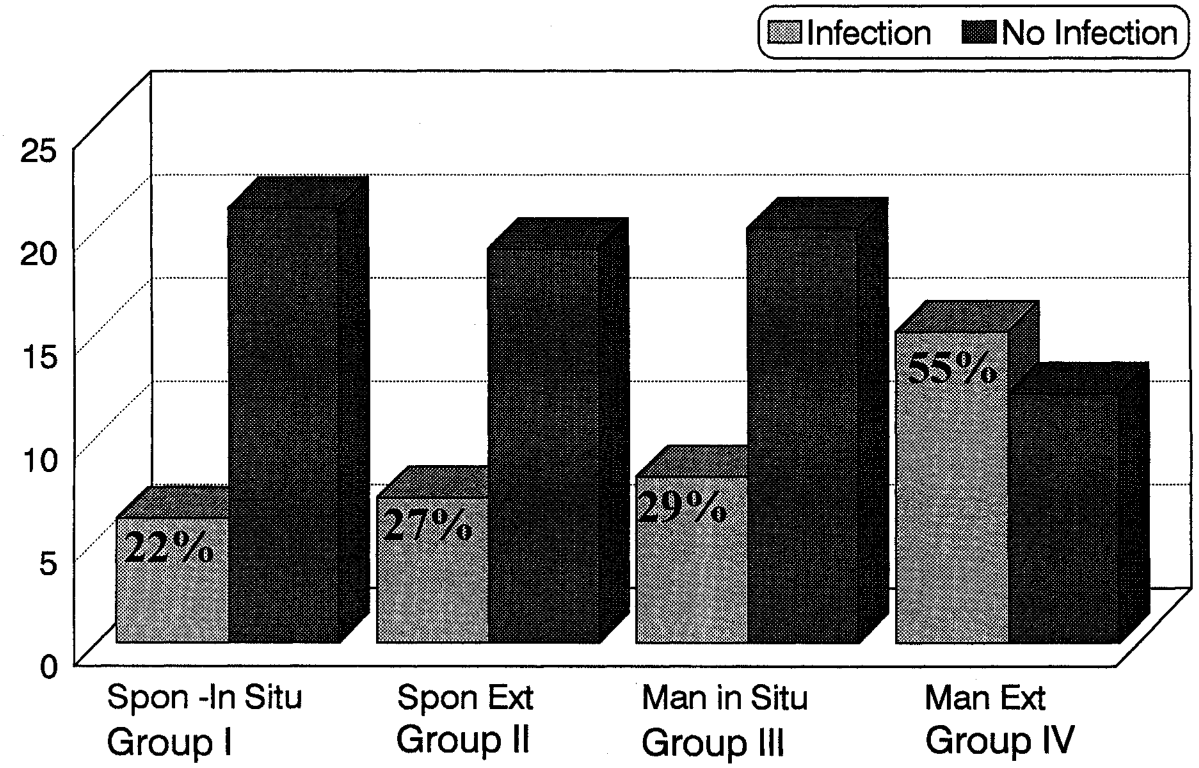

**Fig. 1. Infectious morbidity in each of the four patients groups (group I = 22%, group II = 27%, group III = 29%, and group IV = 55%). Patients with chorioamnionitis were excluded from the study population. $P < 0.005$ for groups I, II, and III compared with group IV. Spon, spontaneous; Man, manual; Ext, exteriorized.**

infected), group I (6 infected, 21 not infected), and group III (8 infected, 20 not infected) produced $P$ values of 0.012, 0.034, and 0.043, respectively. Comparison of similar data in groups I, II, and III by chi-square reflected a $P$ value of 0.9. Since there was no difference among women in those three groups, they can be combined and compared with women in group IV. Comparison of group IV with the combined groups I, II, and III, showed a significant difference between the two populations (relative risk 3.6; 95% confidence interval 1.3–9.7, $P < 0.005$) (Fig. 1).

The duration of labor, length of rupture of membranes prior to delivery, use of an internal monitoring system, and number of vaginal examinations were similar among groups (Table 2). The type of uterine and abdominal incision, the choice of anesthesia, and the expertise of the operating surgeon were not significantly different among the four categories.

Blood loss between groups was significantly increased in the groups with manual removal of the placenta (groups III and IV; 1,342 ± 549 cc and 1,146 ± 280 cc, respectively), compared with the groups with spontaneous expulsion of the placenta (groups I and II; 640 ± 234 cc and 644 ± 235 cc, respectively) ($P < 0.001$). No patient was transfused with any blood products.

Blood cultures were positive in 6% of the patients with postcesarean infectious morbidity and were not significantly different among groups. Two patients did not respond to triple antibiotic therapy and, with a negative CT scan to rule out a pelvic abscess, were started on heparin therapy for suspected septic pelvic thrombophlebitis with resolution of the febrile morbidity.

### DISCUSSION

The most significant finding of this investigation is the demonstration of an increased frequency of postoperative endometritis in women in whom the placenta was manually removed and the uterus exteriorized for repair. Manual placental removal has been shown to increase the rate of postcesarean en-

**TABLE 2. Known factors for infectious morbidity**

| Group | Mean maternal weight (kg) | Mean duration of labor (hr) | Mean duration of rupture of membranes (hr) | Internal monitoring (yes/no) | Mean number of vaginal examinations |
|---|---|---|---|---|---|
| I. Spontaneous in situ | 87.09 ± 19.96 | 13.8 ± 9.4 | 22.7 ± 15.5 | 13/9 | 5.0 ± 3.5 |
| II. Spontaneous exteriorized | 88.45 ± 12.25 | 13.3 ± 8.7 | 14.8 ± 8.9 | 11/11 | 4.7 ± 2.2 |
| III. Manual in situ | 92.99 ± 4.08 | 16.2 ± 8.5 | 15.3 ± 7.7 | 11/11 | 5.6 ± 2.8 |
| IV. Manual exteriorized | 91.17 ± 4.54 | 19.8 ± 17.9 | 18.0 ± 17.2 | 12/8 | 4.5 ± 1.4 |
| *P* value | NS | NS | NS | NS | NS |

NS, not significant.

dometritis even when prophylactic antibiotics were administered.[18] Because cesarean delivery[6] is the most important cause of postpartum infectious morbidity, these findings could influence the reduction of postcesarean infections. Because the number of patients delivered abdominally in this country continues to range between 20% and 25%, a large population of patients with infectious morbidity will have prolonged hospital stays, exposure to antibiotics with potentially serious side effects, and possible long-term consequences on overall maternal health. Many factors contribute to cesarean-related infectious morbidity, including: socioeconomic status of the patient, length of the operative procedure, number of vaginal examinations, maternal weight, amount of operative blood loss, and skill of the operating surgeon.[2–10]

The present study comprises a group of women predominantly of low socioeconomic status. The maternal weight, duration of labor, time of rupture of the membranes prior to operative delivery, number of vaginal examinations, type of uterine and abdominal incisions, and length of the operative procedure were similar among all four groups. However, blood loss was significantly increased in association with manual removal of the placenta (groups III and IV) in comparison with spontaneous placental removal with gentle cord traction (groups I and II, $P < 0.001$), although no patient received blood transfusion. Operative blood loss in excess of 800 ml is associated with an increased frequency of postpartum infections.[9] None of our 120 patients received a blood transfusion, and the group with the greatest mean blood loss (group III) in this study did not have a significantly increased infection rate. Instead, it was the manual removal/exteriorized repair group (group IV), with an average blood loss 200 ml less than that of group III, who had a significant increase in the frequency of postcesarean endometritis.

In contrast to the findings in this investigation, Hershey and Quilligan[16] did not report increased infectious morbidity in women in whom the uterus was exteriorized for uterine incisional repair. Since the method of placental removal was not addressed by those authors, it is unclear whether that was a factor in their findings. We also did not observe increased infectious morbidity unless the placenta was removed manually in addition to exteriorization of the uterus for repair.

Which of the factors of placental removal method or location of uterine repair are most important toward the development of infectious morbidity? If uterine exteriorization alone increases the rate of infection, then the group in which the placenta was spontaneously expelled and the uterus was exteriorized for repair (group II) would be expected to have an increased rate. If manual removal of the placenta alone caused an increased rate of infection, then the rates would have been increased in group III (manual removal of the placenta, in situ uterine repair). We observed increased endometritis only when the placenta was removed manually in combination with uterine exteriorization for repair.

Thus, the findings of this investigation suggest that, when other known causes of endometritis are equal among groups, manual removal of the placenta in association with exteriorization of the uterus for repair significantly increases postcesarean section endometritis.

### ACKNOWLEDGMENT

This work was supported in part by the Vicksburg Hospital Medical Foundation.

### REFERENCES

1. Faro S: Infectious disease relations to cesarean section. Obstet Gynecol Clin North Am 15:685–695, 1988.
2. D'Angelo LJ, Sokol RJ: Time related peripartum determinates of postpartum morbidity. Obstet Gynecol 55: 319–323, 1980.
3. Gibbs RS: Clinical risk factors for puerperal infection. Obstet Gynecol 55:178–183, 1980.
4. Gilstrap LC, Cunningham FG: The bacterial pathogenesis of infection following cesarean section. Obstet Gynecol 53:545–549, 1979.
5. Page FO, Howard PR, Martin JN, Martin RW, Rivlin ME, Morrison JC: High risk factors for cesarean febrile morbidity. J Miss State Med Assoc 28:27–29, 1987.
6. Rehu M, Nilsson CG: Risk factors for febrile morbidity associated with cesarean section. Obstet Gynecol 56:269–273, 1980.
7. Hagen D: Maternal febrile morbidity associated with fetal monitoring and cesarean section. Obstet Gynecol 46:260–262, 1975.
8. Gibbs RS: Infection after cesarean section. Clin Obstet Gynecol 28:697–710, 1985.
9. Haaglund L, Christensen KK, Christensen P: Risk factors in cesarean section infection. Obstet Gynecol 62: 145–150, 1983.
10. Anstey JT, Sheldon GW, Blythe JG: Infectious morbidity after primary cesarean sections in a private institution. Am J Obstet Gynecol 136:205–210, 1980.
11. Nielson TF, Hokegard KH: Postoperative cesarean section morbidity: A prospective study. Am J Obstet Gynecol 146:911–916, 1983.
12. Phelan JP, Clark SL: Cesarean Delivery. New York: Elsevier, pp 201–218, 1988.
13. Cunningham FG, MacDonald PC, Gant NF (eds): Cesarean section and cesarean hysterectomy. In: Williams Obstetrics. 18th ed. New York: Appleton & Lange, pp 451–459, 1989.
14. Dunn LJ: Cesarean section and other obstetric operations. In Scott JR, Disaia PJ, Hammond CB, Spellacy WN (eds): Danforth's Obstetrics and Gynecology. 6th ed. Philadelphia: JB Lippincott, pp 639–658, 1990.
15. Depp R: Cesarean delivery and other surgical procedures. In Gabbe SG, Niebyl JR, Simpson JL (eds): Obstetrics: Normal and Problem Pregnancy, 2nd ed. New York: Churchill Livingstone, pp 635–693, 1991.
16. Hershey DW, Quilligan EJ: Extraabdominal uterine exteriorization at cesarean section. Obstet Gynecol 52:189–192, 1978.
17. Wagensteen GH: The controlled administration of fluid to surgical patients. Minn Med J 25:783–789, 1942.
18. McCurdy CM, Magann EF, McCurdy CJ, Saltzman AK: The effect of placental management of cesarean delivery on operative blood loss. Am J Obstet Gynecol 167: 1363–1367, 1992.
